# Disentangling the Impact of Social Groups on Response Times and Movement Dynamics in Evacuations

**DOI:** 10.1371/journal.pone.0121227

**Published:** 2015-03-18

**Authors:** Nikolai W. F. Bode, Stefan Holl, Wolfgang Mehner, Armin Seyfried

**Affiliations:** 1 Department of Mathematical Sciences, University of Essex, Colchester, United Kingdom; 2 Jülich Supercomputing Centre, Jülich, Germany; 3 University of Wuppertal, Wuppertal, Germany; Nanyang Technological University, SINGAPORE

## Abstract

Crowd evacuations are paradigmatic examples for collective behaviour, as interactions between individuals lead to the overall movement dynamics. Approaches assuming that all individuals interact in the same way have significantly improved our understanding of pedestrian crowd evacuations. However, this scenario is unlikely, as many pedestrians move in social groups that are based on friendship or kinship. We test how the presence of social groups affects the egress time of individuals and crowds in a representative crowd evacuation experiment. Our results suggest that the presence of social groups increases egress times and that this is largely due to differences at two stages of evacuations. First, individuals in social groups take longer to show a movement response at the start of evacuations, and, second, they take longer to move into the vicinity of the exits once they have started to move towards them. Surprisingly, there are no discernible time differences between the movement of independent individuals and individuals in groups directly in front of the exits. We explain these results and discuss their implications. Our findings elucidate behavioural differences between independent individuals and social groups in evacuations. Such insights are crucial for the control of crowd evacuations and for planning mass events.

## Introduction

Crowd evacuations from confined spaces, such as office workers exiting a building during a fire, or football fans leaving a stadium after a bomb threat, provide paradigmatic examples for collective behaviour: local interactions between individuals lead to the global movement dynamics of the crowd [1,2]. In some circumstances and as a starting point it is convenient to assume that individuals interact with all other individuals they encounter in the same way (to avoid collisions, for example; see [3] for a review). However, it quickly becomes apparent that how individuals interact with others can depend strongly on who they are interacting with. Compare, for instance, the scenario of evacuating a large building in the company of strangers or in the company of your parents or children. Would you try to stay close to your family to assist them, if necessary, even if others were overtaking you? Would you pay as much attention to strangers? Small social groups based on kinship or friendships are ubiquitous in human crowds. Therefore, it is important to investigate how the presence of social groups could affect crowd evacuations and conversely how social groups could be affected by crowd evacuations [4–6].

**Fig 1:**
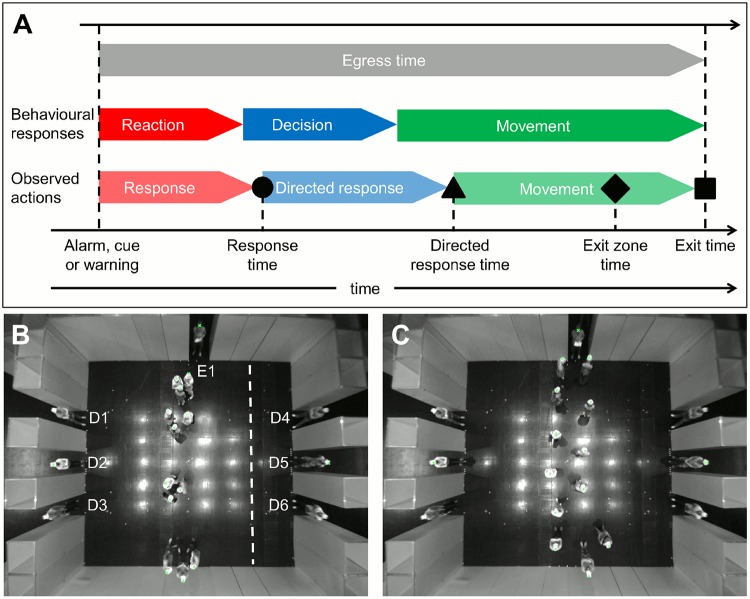
Sequence of behavioural responses during egress and experimental layout. A: diagram for the sequence of behavioural responses of individuals during egress from a room. We show the total egress time in grey at the top. This egress time for individuals comprises a sequence of behavioural responses (second row in A): the time to perceive and interpret an alarm (reaction), the time to make a movement decision (decision) and the time to move out of the room (movement). We cannot directly observe the timing of individuals’ behavioural responses in our experiment. For example, the time at which individuals make a decision on where to move may not coincide with the time they start to move towards their target. We therefore approximate the timings of this behavioural sequence by observing individuals’ actions (‘observed actions’, third row in A), measuring the response time (black circle), directed response time (black triangle) and exit time (black square, see methods). We also measure the time when individuals enter a region directly in front of the exit (exit zone time, black diamond). The response and directed response time may coincide and the relative length of time spent in different behavioural states in A is for illustration purposes only. B and C: layout of the experiment recorded from above with the initial configuration of participants in the group (B) and individual (C) treatment. In B, the entrance through which participants entered the room (E1), the six exits (D1–D6) and the exit zone for D5 (to the right of the dashed line) are marked. In the experiments, only D2 and D5 were opened.

To conduct safe evacuations, it is paramount to understand the factors that impact on the time it takes individuals to reach safety. In addition to the time it takes pedestrians to move along an evacuation route (movement time), this egress time comprises several additional major components linked to individuals’ behavioural responses (e.g. pre-movement time; [7]). Fig. 1A shows a simplified diagram illustrating the sequence of behavioural responses individuals show during an evacuation (adapted from [7]). Individuals have to perceive and subsequently interpret new information when an alarm goes off or a warning is issued. This results in a time delay between the alarm and individuals’ reaction. Once individuals are aware of the situation, they have to decide on what action to take. If individuals decide to evacuate, they often have to choose between different available exit routes. To inform their decision, individuals may also attempt to acquire additional information. This decision making process results in an additional time delay before individuals start to move towards safety along their chosen evacuation route. In Fig. 1A, the reaction and decision processes described above combine into a pre-movement time, but depending on the context, individuals may undergo similar behavioural processes multiple times during an evacuation. For example, individuals may have to react to additional information that only becomes available when they are already moving or individuals may have to adjust their exit route choice when preferred exits become crowded.

A key aspect of research into crowd evacuations is to develop an understanding of how different circumstances affect these major components of egress time [3]. For example, obstacles, exit widths or increasing numbers of evacuees can affect the movement speed of individuals [3,8] and recent work has investigated the effect of motivation levels or stress on exit route choices during evacuations [9]. Human crowds are rarely composed exclusively of unrelated and independently moving individuals. Instead, crowds often contain many small social groups based on friendship or kinship [10]. Even if individuals within a crowd are not socially connected, the experience of an emergency may lead to a shared identity connecting individuals in a similar way to social groups [5]. In the following we discuss how the presence of social groups could affect all major components of the egress time introduced above.

The processes involved in responding to an alarm and in making movement decisions are different in social groups compared to individuals. This could be a disadvantage for members of social groups. Empirical observations suggest that individuals seek the proximity of familiar people under adverse conditions ([11] and references therein). Therefore, dispersed social groups may initially seek to assemble in response to an alarm, causing a delay in their evacuation. Making movement decisions is also likely to take longer in social groups. If group cohesion is to be maintained, individuals need to reach consensus on their movement direction by one of a number of group decision making processes (e.g. [12,13]). To this end, individuals have to communicate or even discuss their alternatives vocally, through gestures or by moving in their preferred direction [12,13], thus possibly incurring a time delay.

However, making decisions in a group may also be advantageous in evacuations. Previous research has shown that by pooling incomplete individual information, groups can make better or more accurate decisions than individuals under some circumstances (see e.g. [14] for a review). A simple example could be that two members of a group combine their information about alternative exit routes that will reduce the overall egress time of the group. Social groups may also outperform individuals in acquiring information. For example, it has been shown that groups are better than individuals at reconstructing the content of loudspeaker announcements in noisy environments [15]. Decisions based on accurate information are likely to lead to more efficient evacuations. Experiments with volunteers have also shown that social groups can react faster to ambiguous information than groups of unfamiliar people [16–18]. In such situations social connections or familiarity can help to overcome peoples' inherent reluctance to stand out by being the first to respond, potentially falsely, to an ambiguous cue.

The specific context determines whether and to what extent the different aspects discussed above affect the egress times of individuals in social groups. While invaluable insights have been gained from analysing eye witness reports of real emergencies [11,19], we suggest that further controlled experiments that simulate representative evacuation scenarios are needed to gauge the effect social groups have on individuals' pre-movement times and movement decisions in crowd evacuations.

The presence of social groups could also impact on the movement dynamics of evacuating crowds. Empirical evidence shows that social groups of pedestrians adopt particular formations and it has been suggested that this facilitates communication between group members [10]. Based on eye witness reports it has been proposed that members of social groups and in particular of families display strong affiliative behaviour and stay close to each other even under severe environmental threat [11]. Intuitively, these observations suggest that social groups could effectively form moving units which may turn into moving obstacles within pedestrian crowds. To test this intuition, social groups have been included in individual-based models for pedestrian movement by assuming that group members seek to maintain particular group formations (e.g. a line or an inverted arrow head) or at least remain close to each other [4,6,10,20]. Computer simulations of such models suggest that the presence of social groups can affect the flow of pedestrians in corridors. Depending on the particular assumptions of how individuals in social groups interact, this can result in more efficient [20] or less efficient [6] crowd movement. When considering pedestrian crowds passing through bottlenecks, such as doors, the evidence is similarly ambiguous and depends on how interactions between individuals in social groups are implemented [4,6,21]. The presence of social groups could result in conflicts at exits between entire groups on who can exit first. Such conflicts may be more difficult to resolve than similar conflicts between individuals and may result in entire groups waiting until other groups have exited. Experimental tests of such hypotheses are lacking to date. The few experiments that have been conducted suggest that the presence of social groups increases the egress time from a room [6]. However, the authors only report egress times. It is therefore necessary to conduct experiments that allow us to disentangle the origin and mechanisms behind the increase in egress time in the presence of social groups in more detail. Can the differences in egress time be explained by altered crowd movement dynamics in the presence of social groups? Or are the differences largely due to pre-movement or decision making processes that are specific to social groups?

Here we report the findings of an experiment that simulates a representative evacuation scenario with volunteers. The key novelty and strength of our approach is that our experimental design ensured that participants followed a sequence of separate behavioural responses that can be used to estimate the duration of the major components of the egress time introduced above and in Fig. 1A. Comparing the timing of these behavioural responses between crowds of independently moving individuals and crowds composed of social groups allowed us to pinpoint which components of the egress time were affected by the presence of social groups. Our findings confirm some of the scenarios introduced above, but they also raise important questions for future research.

## Methods

### Experimental design

Experiments took place on the 21^st^ of June 2013 in Düsseldorf, Germany. We conducted experiments with groups of 12 pedestrians in a square room (10x10m) with six exits, three each on two opposite sides of the room, and one entrance positioned centrally on a third side of the room. We filmed the experimental setup from above (see Fig. 1B,C). All six exits were 1m wide and initially blocked by helpers who were unaware of the precise purpose of the experiment. Participants entered the room through the separate entrance and were positioned along the line equidistant from the two sides of the room where the exits were situated (see below for more details on positioning). Subsequently, participants received the following instructions (in German, translated here): *‘You are in a room with six exits*. *At the moment all exits are blocked*. *When I shout GO*, *some of the exits will be opened*. *When this happens*, *leave the room through any of the open exits*.*’* The experimenter then provided additional instructions particular to the experimental treatment (see below), blocked the entrance by standing in it and waited for 20–30s before starting the experiment with the ‘GO’ command. On this command the helpers blocking the two central exits stepped aside and the participants left the room through these exits.

We split a total of 120 participants into 10 separate groups by asking 12 participants at a time to come forward from a waiting area. Each of the participant groups was subjected to two experimental treatments. The experimental treatments were designed to approximate the situation when pedestrians belong to small social groups, such a friendship groups or families (‘group treatment’) and the situation when all pedestrians move independently (‘individual treatment’). After experiencing both experimental treatments, participant groups left the experimental setup and went to another waiting area that was separate from the first waiting area. We alternated the order in which the treatments were applied between consecutive groups to obtain a balanced experimental design and ensured that participants who had not yet taken part could not see the layout of the experimental setup. The treatments were ordered as follows: the first participant group experienced the group treatment first, the second group experienced the individual treatment first, the third group experienced the group treatment first, and so forth.

The instructions to participants and their placement within the experimental room at the start of the experiment differed between the two treatments: in the ‘group treatment’, participants were split up into groups of three participants each which were positioned along the central line described above (Fig. 1B). They received the following instructions: *‘We have split you up into groups*. *Please stay together in these groups until you have left the room*. *Before I shout GO*, *I would like you to talk about your favourite food within your group’*. These instructions had the purpose to model communication and cohesion of natural social groups. We set the size of social groups to three pedestrians to ensure our experiment approximated the behaviour of typical social groups larger than pairs of pedestrians (see e.g. group sizes observed in [10]). In general, typical social group sizes are likely to depend on the context and we further discuss the possible effects of the size of social groups below.

In the ‘individual treatment’, participants were positioned individually along the central line described above (Fig. 1C). They were then told: *‘When I shout GO*, *please leave the room independently from each other*. *Do not wait for others*. *Before I shout GO*, *I would like you to start quietly to count upwards from 1 to 1000*.*’* Participants were told to count quietly to introduce a distraction comparable to the conversation initiated in the group treatment. By asking selected participants to swap starting positions, we endeavoured to ensure that participants who appeared to have arrived for the experiment in friendship groups did not occupy nearby starting positions in the individual treatment.

All procedures of our experiment were approved by the Ethics Committee of the University of Essex. Informed consent of participants was obtained in written form.

### Data collection and analysis

We filmed the experiments directly from above at a frequency of 16 frames per second. A technical fault meant that the experiment of the last participant group was not recorded. All results are therefore for 9 participant groups and a total of 108 participants. From the videos we extracted the position of each participant and of the helpers who blocked exits D2 and D5 in two dimensions. We used a semi-automated procedure to track the heads of individuals as follows. Initially, the positions of the centre of individuals' heads were determined manually in one frame of the video recording. These positions provided the starting points for individuals' trajectories which were tracked automatically in subsequent frames, using a well-established computer vision technique (KLT feature tracking; [22]). We visually checked the results of this procedure and manually corrected the trajectories, where necessary. The locations of the exits D2 and D5 were also obtained directly from video frame images. The positions of individuals in the experimental room were re-scaled from image pixels to metres using the known distance between the mid-points of exits D2 and D5. Individual trajectories were smoothed by using a moving average over a window of 5 frames. Speeds were defined as the displacement between two consecutive positions in the smoothed trajectories (1/16s apart).

Although the experiments were initiated by a ‘GO’ command, it was the movement of the helpers blocking the doors that informed participants about the exit options available. We therefore defined the starting point of our experiments by using the time point when the helpers blocking the exits started to move. Specifically, we recorded the time points at which each helper’s speed exceeded a fixed threshold. We chose a high speed threshold of 0.75m/s to avoid falsely recording spurious head or body movements as the start of the experiment. As the helpers’ reaction was apparent before they reached the speed threshold, we used the time points 8 frames ( = 0.5s) before the speed threshold was reached in our analysis. The two helpers did not always start to move at the same time. However, the mean difference between the two helpers was low at 0.21±0.09s (mean±standard error) and we found no consistent bias in the helpers’ movement start times (Wilcoxon signed-rank test, W = 58.5, P = 0.95). Therefore, we used the first reaction time of the two helpers as the starting point of our experiment. At this starting point, we measured the average distance of individuals to their nearest exit for each experimental run to ensure that the different initial positioning of participants in the two treatments did not introduce a bias (approximating exit locations by the midpoint of the 1m wide exits D2 and D5). We found no significant difference between the two treatments (groups: 5.16±0.05m; individuals: 5.05±0.05m; mean±s.e.; Wilcoxon signed-rank test, W = 33, P = 0.25).

Within our experimental setup, we cannot determine the precise time-points of when individuals reacted to the ‘GO’ command or when individuals decided which exit to use. Instead, we approximate the timings of these behavioural responses by observing individuals’ actions. For example, we record the time at which individuals start to move which approximates, but is unlikely to coincide with the time it takes individuals to perceive and interpret the ‘GO’ command. The summary statistics we extracted from the trajectory data to quantify participants’ behaviour are described in the following (see also Fig. 1A).

*‘Response time’*: we defined the response time of an individual to be the time point at which the speed of the individual exceeded a fixed threshold of 0.75m/s. We chose a relatively high threshold to avoid errors from spurious movement, such as head turns or changes of the supporting leg whilst standing in one place. In the context of pedestrian evacuations, the time individuals show a movement response is important. If it takes individuals longer to start moving, this may reflect that they are contemplating different routes or that the evacuation signal was ambiguous. We computed the average response time for each experimental run.

*‘Directed response time’*: we defined the directed response time of individuals to be the time point at which the component of their speed which was directed towards their final exit exceeded the fixed threshold of 0.75m/s. For this purpose, the two exits were approximated by points located centrally in the 1m wide exit and we computed the dot product between individuals’ velocity vector and the unit direction vector pointing from individuals’ position towards the exit points. The directed response time of individuals is informative, as some individuals may start to move before having made a final decision on which exit to use. In social groups, initial movement may even be used to communicate movement preferences to other group members. Furthermore, individuals may change their mind on which exit to use in the course of the experiment. Such decision changes could result either from a change of the preferred exit (e.g. originally preferred exit is over-used) or from observing the movement decisions or changes in movement decisions of other group members. While the former process can take place in individuals and groups, the latter process is limited to social groups. Response and directed response times may coincide if individuals start to move directly towards the exits they end up using. We computed the average directed response time for each experimental run. Participants were stationary at the start of the experiment and our approach captured clearly visible changes in movement in a consistent way (see results below).

*‘Exit zone time’*: To distinguish between time spent moving towards and standing in a jam nearby the exits, we measured the time point when individuals entered their ‘exit zone’, a region that started 2m before the wall containing the exit used by individuals (see Fig. 1B). We report the average exit zone time for each experimental run. This measure allowed us to localise differences between the two experimental treatments.

*‘Average exit time’* and *‘Last exit time’*: For each individual we determined the time point at which they crossed the line segment defined by the exit they used. From the individual exit times we computed the average exit time for each experimental run and we also recorded the time point when the last participant exited the room, as both the total time to empty the room (last exit time) and the average exit time across all individuals is of interest.

These summary statistics describe a clear sequence of events: participants show a movement response (response time), later or simultaneously their movement is directed towards the exit they end up using (directed response time), then they enter the area immediately in front of the exit (exit zone time) and finally they exit the room (exit time). An important aspect of our analysis is that we considered the length of the time intervals between these events.

### Statistical analysis

We conducted our statistical analysis in the R programming environment, version 3.0.2 [23]. To test the statistical significance of the difference in summary statistics between the two experimental treatments, we used Wilcoxon signed-rank tests. Throughout, we report the test statistic, W, and the p-value, P. Whenever other statistical tests were used we specify them explicitly in the text. We set a significance threshold of P<0.05, but we report and discuss the p-values of all statistical tests. Because of the balanced design of our experiment, we did not consider treatment order in our statistical analysis.

To test whether unbalanced exit use (e.g. 9 people use exit D2 and 3 people use D5 in Fig. 1B) had an effect on the time interval between the directed response and the exit time, we used a likelihood ratio test on fits of Linear Mixed Models (LMMs) to the data. LMM fits were obtained using the ‘nlme’ package in R [24]. The full model included the time interval between the directed response and the exit time for each experimental run as the response variable (n = 18). The explanatory variables were treatment (categorical, levels: ‘individual’ and ‘group’) and our measure for imbalance of exit use (defined below). A fixed effect intercept, as well as a random intercept for participant groups (9 separate groups) were also included in the model. In the likelihood ratio test we compared the full model to a reduced model that did not include the imbalance measure as an explanatory variable.

## Results

There was no consistent bias in the exits participants chose in our experiments. First, we tested whether the proportion of participants or groups using exit D2 (see Fig. 1B) across all 9 participant groups was significantly different to what we would expect by chance. This was neither the case in the individual treatment (52 out of 108 participants used exit D2; binomial test, P = 0.77) nor in the group treatment (21 out of 36 groups used exit D2; binomial test, P = 0.41). In the group treatment, no social group split up between the exits. Second, we investigated whether either treatment led to a larger imbalance between exits. For this, we computed the fraction of participants using the more popular exit for each experimental run (between 0.5 and 1.0). We found that this measure of imbalance was 0.64±0.044 (mean±s.e.) for the group treatment and 0.57±0.022 (mean±s.e.) for the individual treatment. The difference between experimental treatments corresponds to less than one participant and was not statistically significant (W = 23, P = 0.14).

**Fig 2:**
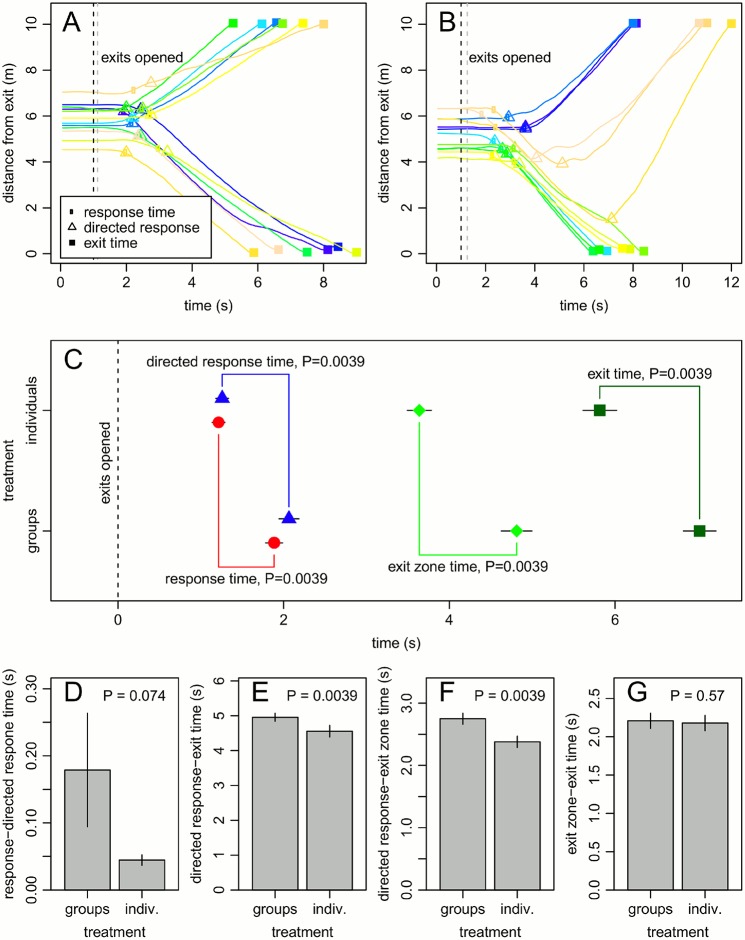
Experimental results. A,B: distance of participants from the centre of exit D2 over time. A and B show the data for different participant groups for the individual and the group treatment, respectively. The black vertical dashed line shows the starting point of the experiment (the corresponding time for the helper reacting slower is shown in grey, see methods). Panel C shows the average response, directed response, exit zone and exit time across 9 participant groups for the two experimental treatments. These times approximately quantify the sequence of behaviours individuals undergo (compare to Fig. 1A). D-G show the average lengths of time intervals between the time points measured in C. Error bars in C-G show standard errors. P-values are from Wilcoxon signed ranks tests (see methods and main text for full details). Identical P-values for varying differences in means arise from the fact that the test statistic is based on the ranking of differences and does therefore not depend on the size of the difference.

Fig. 2A,B show output of our analysis of the trajectories. While the response and directed response times of individuals often coincided, there were also cases when the directed response occurred considerably later than the first response. We also observed changes in individuals’ exit choice during experimental runs (see Fig. 2B).

Across participant groups, we found that the average and last exit times in the group treatment were significantly higher than in the individual treatment. The average exit time was 7.02±0.20s (mean±s.e.) in the group treatment and 5.82±0.20s (mean±s.e.) in the individual treatment, a statistically significant difference (W = 45, P = 0.0039; Fig. 2C). The total time to empty the room, or last exit time, was 9.25±0.36s (mean±s.e.) in the group treatment and 7.85±0.27s (mean±s.e.) in the individual treatment (W = 36, P = 0.014). This shows that the differences in exit times between treatments were not big in absolute terms (just over 1s). However, this has to be viewed in relation to the overall time it took to empty the room, a maximum of less than 10s, implying an increase in exit time of over 10%.

There are a number of factors that could explain the differences in exit times between the two treatments. First, group response times and decision making processes could affect the time it takes individuals to make movement decisions in groups. For example, in the introduction we discussed how reaching consensus in a group may take longer than it takes an individual to make a movement decision. In the context of our analysis, we would expect such differences to be reflected in the response times (if groups make decisions prior to moving) or the directed response times (if groups make decisions on the move), or both. Second, unbalanced and therefore suboptimal use of the two exits could result in larger exit times. Although the difference between treatments was not statistically significant, our measure for imbalance was slightly higher for the group treatment which could explain part of the difference in exit times between the two treatments. Third, the treatments could directly affect the movement of people at the exits. This could arise from ‘conflicts’ between groups at exits, when entire groups wait until another group has exited, for example. Fourth, individuals’ decisions could differ between the treatments. For example, independent individuals may be more likely than members of groups to choose the exit closer to them as they are not restrained by other group members. To disentangle the effects of these different factors we consider them in turn whilst accounting for the variance in exit times explained by other factors.

We found that both response and directed response times differed significantly across treatments (Fig. 2C). The average response time decreased from 1.88±0.10s (mean±s.e.) in the group treatment to 1.21±0.08s (mean±s.e.) in the individual treatment (W = 45, P = 0.0039). Likewise, the average directed response time showed a statistically significant decrease from the group treatment to the individual treatment (2.06±0.12s and 1.26±0.08s, respectively; W = 45, P = 0.0039). In other words, it took individuals in the group treatment longer to show a movement response which may be due to pre-movement decision making processes in groups. If social groups finalised decisions predominantly whilst already moving (e.g. if individuals indicated their preferences by moving towards an exit), we would expect that the time interval between response times and directed response times is higher under the group than under the individual treatment. We found that this was the case, but that the difference was not statistically significant (Fig. 2D; groups: 0.18±0.09s; individuals: 0.05±0.01s; W = 38, P = 0.074). The p-value, although not significant, is still quite low and the variance of the response to directed response time intervals in the group treatment is large. This large variance is the result of some groups initially moving off in one direction but then changing their mind (see e.g. Fig. 2B). However, the difference in response to directed response time intervals between treatments was rather low and our results thus show that the difference in response times accounts for most of the difference in directed response times between treatments.

By comparing the length of the time interval between the directed response and the exit time across treatments, we can account for the variance in exit times introduced by processes that occur prior to directed movement towards an exit. We found that this time interval was significantly lower in the individual treatment compared to the group treatment (Fig. 2E; groups: 4.96±0.12s; individuals: 4.56±0.17s; W = 45, P = 0.0039). In other words, once every participant showed a clear indication of her final movement decision, it still took participants on average longer to exit the room in the group treatment. As discussed above, this could be explained by the less balanced exit use in the group treatment. However, we found no significant effect of our measure for imbalance on the length of the time interval between directed response and exit times (likelihood ratio test: X^2^(1) = 0.23, P = 0.63).

To localise where the difference between the treatments in directed movement towards the exits occurred, we considered the exit zone time. This allowed us to distinguish between movement directly in front of the exits and movement towards the exit zone. The time interval between the directed response time and the exit zone time differed significantly between treatments (Fig. 2F; groups: 2.75±0.09s; individuals: 2.38±0.09s; W = 45, P = 0.0039). In contrast to movement towards the exits, the time interval between the exit zone time and the exit time did not differ between treatments (Fig. 2G; groups: 2.21±0.10s; individuals: 2.18±0.10s; W = 28, P = 0.57). This suggests that groups took longer to move towards the exit zone, but once participants were directly in front of an exit, moving in a group did not affect individuals’ exit times.

The average speed of individuals when moving towards the exit zone (time interval between the directed response time and the exit zone time) did not vary significantly between treatments (groups: 1.18±0.03m/s; individuals: 1.24±0.04m/s; W = 10, P = 0.16). This suggests that members of social groups followed longer movement paths towards the exits. Indeed, the average distance of individuals to their chosen exit at the directed response time was significantly higher for groups (5.28±0.08m) than for individuals (5.04±0.07m; W = 40, P = 0.039). In part, this difference in movement path lengths can be attributed to the fact that in the group treatment, participants were slightly more likely to move towards one exit before changing their mind and exiting through the opposite exit (see e.g. Fig. 2B). This is indicated by the fact that the time interval between response times and directed response times is higher under the group than under the individual treatment (Fig. 2D). Another contributing factor to the difference in movement path lengths is the fact that in the individual treatment participants were more likely to exit through the exit nearer to them than in the group treatment. We compared the fraction of individuals who exited through their nearest exit between the individual (0.75±0.05) and the group treatment (0.59±0.05; W = 27, P = 0.034) and tested the effect of this fraction on the length of the time interval between the directed response time and the exit zone time (likelihood ratio test, similar to above: X^2^(1) = 5.15, P = 0.023). If we assume individuals tend to choose the exit nearer to them, this difference between treatments makes sense intuitively: in our experiment group members are arranged around the central line in the room, equidistant from both exits (Fig. 1B). It is therefore impossible for all group members to exit through their nearest exit. Independent individuals are not limited in this way. Staying in their group can thus be a disadvantage for some group members, as it increases the distance they have to walk to reach an exit.

## Discussion

We conducted an experiment to test which major components of the egress time are affected by the presence of small social groups in a representative crowd evacuation scenario. We found that the presence of social groups increased the average egress time of individuals. This difference in egress time was largely due to differences in two major components of the egress time. First, individuals in social groups took longer to show a movement response and second, they took longer to move into the vicinity of the exits once they had started to move towards them. There were no discernible time differences between the movement dynamics of independently moving individuals and individuals in groups directly in front of the exits and in the interval between the first movement response and the directed movement response towards one of the exits.

Our findings show that members of social groups did not indicate their movement preferences by quickly moving towards an exit. Had this been the case, we would have expected there to be a significant difference in the time interval between the first movement response and the directed movement response between the group and the individual treatment. Therefore, movement decisions in social groups were typically made before individuals started to move (although there were some exceptions, see Fig. 2B) and this pre-movement decision making process took longer in groups than individuals. This is plausible, considering that movement preferences have to be communicated between group members [12,13]. It is possible and perhaps even likely that in addition to vocal communication, group members used subtle movements, such as turning towards an exit, to indicate their movement preferences (e.g. [25]). However, the data we collected did not allow for such a detailed investigation of pre-movement decision making processes in social groups.

Previous studies testing the effect of social groups on crowd evacuations have either focussed on the movement dynamics directly in front of exits [4], or have not distinguished explicitly between movement towards and at the exits [6]. We distinguished between these two types of movement dynamics. In our experiments there is no discernible time difference in the movement dynamics in front of the exits. Instead, most of the difference between the group and individual treatment arises during movement towards the exits. We suggest that a combination of factors explains this difference in time taken to move towards the exits in our experiment. Differences between treatments in walking speeds and the frequency of changes in exit choice play a smaller role and independent individuals choosing their nearest exit more frequently plays a significant role. However, it is likely that the relative importance of these different factors depends on the specific context.

At an exit, pedestrians have to establish the order in which they exit. This can impede the rate at which individuals exit. An everyday example is given by the situation when two individuals reach an exit at the same time and both stop and wait for the other to exit first. If individuals are positioned some distance away from an exit, they can establish the order in which they exit on their approach towards the exit. If individuals are positioned directly in front of an exit, they cannot establish their exiting order on the move. It is possible that the number of conflicts on the order in which people exit, similar to the example above, could be different between these two scenarios. Experiments with independently moving individuals have shown that whether or not individuals are positioned directly in front of an exit at the start of experiments only has a minor effect on the rate at which individuals exit [3]. Giving independent individuals the opportunity to decide the order in which they exit on their approach towards an exit does therefore not appear to improve the flow of pedestrians through exits. Above, we hypothesised that there could be conflicts on the exiting order between entire social groups directly in front of exits and that such conflicts may not be as easily resolved as similar conflicts between individuals. Our results suggest that such conflicts, if they occurred, did not cause time delays at exits. Instead, social groups may have established their exiting order on the approach towards the exits. However, these observations have to be viewed in the context of the relatively small number of participants involved in our experiments. A maximum of three social groups used the same exit in our experiments which resulted in a rather low number of potential conflicts between groups at exits. At this stage we therefore caution against ruling out an effect of social groups on pedestrian movement dynamics at exits when compared to independently moving individuals. More generally, the number of individuals at an exit or bottleneck is known to be one of the key aspects affecting the flow of pedestrians [3,8]. Therefore, we suggest further experiments testing the effect of social groups on evacuation dynamics with varying crowd densities are needed.

While we did not test this here, it is likely that the time delays incurred by decision making processes and the movement dynamics of social groups depend on the size of the social groups. Indeed, one study has shown that the egress time of a crowd increases with the size of the social groups that the crowd is divided into [6]. More specifically, the time it takes social groups to reach a consensus decision on movement may also increase with group size in many cases [13]. In addition, social groups and independent individuals or social groups of different sizes may interact in different ways. For example, a group may stop to let an individual exit first. This could be tested in experiments with heterogeneous groups that include independent individuals as well as social groups of different sizes. It is possible that in many large-scale experiments on pedestrian dynamics (e.g. [8,26]), the participant recruitment mechanisms naturally lead to such a heterogeneous crowd composition, as friendship groups are likely to sign up together for experiments. As a starting point, it would be interesting to establish to what extent this is the case and to what extent friendship groups tend remain cohesive during such experiments. Explicitly conducting experiments that only include independent individuals or social groups of a certain size, as we have done here, may therefore be particularly useful, as it establishes an understanding of egress under the extremes of homogeneous crowd composition. We might expect that the egress behaviour of heterogeneous crowds lies in between these extremes.

Crowd evacuations are often conducted under real or perceived time pressure which will affect the behaviour of evacuees [27]. For example, a computer-based experiment on human decision-making suggests that the stress associated with increased time pressure makes individuals less likely to adjust initial movement decisions in the course of a simulated evacuation [9]. Considering social groups, observational data from a real emergency show that stress can lead to stronger affiliative behaviour between familiar individuals [11]. This highlights the importance of investigating the effect of time pressure or stress on the behaviour of social groups at different stages of evacuations (e.g. decisions, movement). In particular, it would be interesting to determine whether stress or time pressure exacerbates or reduces the differences between independent individuals and social groups in evacuations.

Our findings show in detail how being in a social group can affect the pre-movement time and the movement time of evacuating individuals. We suggest that this has implications for the design of simulation models that are used to aid the design of buildings (for a review of such models, see [3]). For example, it is not enough to account for social groups in the movement dynamics alone, as some models already do [4,6,20], but we also need to take into account how social groups make movement decisions and how long this takes (e.g. decisions on when to move and where to move if there are multiple options, or even conflicting preferences within groups). As mentioned in the introduction, such decision making processes could take place multiple times during evacuations which highlights the need to improve our understanding of this social aspect of crowd evacuations. In Table 1 we highlight the main outcomes of our work and make recommendations for future work. It may be that different evacuation protocols are appropriate, depending on the layout of buildings and the composition of crowds. If this was the case, tools to detect social groups in crowds [28,29] or to determine the extent to which individuals interact socially [30] will be useful to select appropriate evacuation strategies in particular contexts.

**Table 1:** Outcomes and recommendations for future work.

Aspect of evacuation	Outcome/Recommendation for future work
Pre-movement time	If multiple exit routes are available, social groups are likely to take longer to reach a movement decision.
Movement towards exits	Group members will accept suboptimal route choices (e.g. longer routes) to maintain group cohesion (see also [4] for groups that are not assembled initially).
Movement towards exits	Further research is needed into social groups' movement decisions 'on the move'. Our data suggests that while on average there is no difference between individuals and groups, the behaviour of social groups is more variable (see Fig. 2D).
Movement in front of exits	Further research into interactions between groups and between groups and independent individuals in front of exits is needed (e.g. effect of politeness).

This table focuses on direct outcomes of the research presented here and is therefore not an exhaustive list of the effect social groups can have on crowd evacuation dynamics.

## Supporting Information

S1 Dataset Dataset of individual movement trajectories. Zip archive containing separate files for different experimental runs. Please refer to the file README.txt inside the Zip archive for further information on how the data is presented.
(ZIP)

## References

[1] Camazine S, Deneubourg J-L, Franks N, Sneyd J, Theraulaz G, Bonabeau E. Self-organization in biological systems. Princeton, NJ: Princeton University Press; 2001.

[2] Couzin ID, Krause J. Self-organization and collective behavior in vertebrates. Adv Stud Behav. 2003;32: 1–75.

[3] Schadschneider A, Klingsch W, Kluepfel H, Kretz T, Rogsch C, Seyfried A. Evacuation Dynamics: Empirical Results, Modeling and Applications In: Meyers RA, editor. Encyclopedia of Complexity and Systems Science. Heidelberg: Springer; 2009 pp. 3142–3176.

[4] Braun A, Musse SR, de Oliveira LPL, Bodmann BEJ. Modeling individual behaviors in crowd simulation. In: Proceedings of the 16th International Conference in Computer Animation and Social Agents. IEEE; 2003. pp. 143–148

[5] Drury J, Cocking C, Reicher S. Everyone for themselves? A comparative study of crowd solidarity among emergency survivors. Br J Soc Psychol. 2009;48: 487–506. 10.1348/014466608X357893 18789185

[6] Köster G, Seitz M, Treml F, Hartmann D, Klein W. On modelling the influence of group formations in a crowd. Contemp Soc Sci. 2011;6: 397–414.

[7] Proulx G. Movement of people: the evacuation timing In: DiNenno PJ, editor. SFPE Handbook of Fire Protection Engineering. 3rd edition Quincy, MA: National Fire Protection Association; 2002 pp. 343–366.

[8] Seyfried A, Rupprecht T, Passon O, Steffen B, Klingsch W, Boltes M. New insights into pedestrian flow through bottlenecks. Transport Sci. 2009;43: 395–406.

[9] Bode NWF. Codling EA. Human exit route choice in virtual crowd evacuations. Anim Behav. 2013;86: 347–358.

[10] Moussaïd M, Perozo N, Garnier S, Helbing D, Theraulaz G. The walking behaviour of pedestrian social groups and its impact on crowd dynamics. PloS ONE 2010;5: e10047 10.1371/journal.pone.0010047 20383280 PMC2850937

[11] Sime JD. Affiliative behaviour during escape to building exits. J Environ Psychol. 1983;3: 21–41.

[12] Conradt L, List C. Group decisions in humans and animals: a survey. Proc R Soc B. 2009;364: 719–742. 10.1098/rstb.2008.0276 PMC2689721 19073475

[13] Sumpter DJ, Pratt SC. Quorum responses and consensus decision making. Phil Trans R Soc B. 2009;364: 743–753. 10.1098/rstb.2008.0204 19073480 PMC2689713

[14] Krause J, Ruxton GD, Krause S. Swarm intelligence in animals and humans. Trends Ecol Evol. 2010;25: 28–34. 10.1016/j.tree.2009.06.016 19735961

[15] Clément RJG, Krause S, von Engelhardt N, Faria JJ, Krause J, Kurvers RHJM. Collective cognition in humans: groups outperform their best members in a sentence reconstruction task. PLoS ONE 2013;8: e77943 10.1371/journal.pone.0077943 24147101 PMC3798465

[16] French JRP. Organised and unorganised groups under fear and frustration. U Ia Stud Child Welf. 1944;20: 229–308.

[17] Latane B, Darley JM. Group inhibition of bystander intervention in emergencies. J Pers Soc Psychol. 1968;10: 215–21. 5704479 10.1037/h0026570

[18] Rutkowski GK, Gruder CL, Romer D. Group cohesiveness, social norms, and bystander intervention. J Pers Soc Psychol. 1983;44: 545–552.

[19] Johnson CW. Lessons from the evacuation of the World Trade Center, Sept 11th 2001, for the future development of computer simulations. Cogn Technol Work. 2005;7: 214–240.

[20] Qiu F, Hu X. Modeling group structures in pedestrian crowd simulation. Simul Model Pract Th. 2010;18: 190–205.

[21] Yang LZ, Zhao DL, Li J, Fang TY. Simulation of the kin behavior in building occupant evacuation based on cellular automaton. Build Environ. 2005;40: 411–415.

[22] Shi J, Tomasi C. Good features to track. In: Proceedings IEEE Computer Society Conference on Computer Vision and Pattern Recognition. IEEE; 1994. pp. 593–600.

[23] R Core Team. R: A language and environment for statistical computing. Vienna, Austria: R Foundation for Statistical Computing; 2013 Available: http://www.R-project.org/. 10.3758/s13428-013-0330-5

[24] Pinheiro J, Bates D, DebRoy S, Sarkar D, R Development Core Team. nlme: Linear and Nonlinear Mixed Effects Models R package version 3.1–113. Vienna, Austria: R Foundation for Statistical Computing; 2013 Available: http://www.R-project.org/.

[25] vom Lehn D. Withdrawing from exhibits: The interactional organisation of museum visits In: Haddington P, Mondada L, Neville M, editors. Interaction and Mobility: Language and the Body in Motion. Berlin, Germany: De Gryter; 2013 pp. 65–90.

[26] Hoogendoorn SP, Daamen W. Pedestrian behavior at bottlenecks. Transp Sci. 2005;39: 0147–0159

[27] Ozel F. Time pressure and stress as a factor during emergency egress. Safety Sci. 2001;38: 95–107.

[28] Šochman J, Hogg DC. Who knows who-inverting the social force model for finding groups. In: IEEE International Conference on Computer Vision Workshops. IEEE; 2011. pp. 830–837.

[29] Ge W, Collins RT, Ruback RB. Vision-based analysis of small groups in pedestrian crowds. IEEE T Pattern Anal. 2012;34: 1003–1016. 10.1109/TPAMI.2011.176 21844622

[30] Bode NWF, Franks DW, Wood AJ, Piercy JJB, Croft DP, Codling EA. Distinguishing social from non-social navigation in moving animal groups. Am Nat. 2012;179: 621–632. 10.1086/665005 22504544

